# Chasing Metaverses: Reflecting on Existing Literature to Understand the Business Value of Metaverses

**DOI:** 10.1007/s10796-022-10364-4

**Published:** 2022-12-24

**Authors:** Ariana Polyviou, Ilias O. Pappas

**Affiliations:** 1 Department of Management, School of Business, University of Nicosia, 2417 Nicosia, Cyprus; 2 Department of Information Systems, University of Agder, Universitetsveien 25, 4604 Kristiansand, Norway; 3 Department of Computer Science, Norwegian University of Science and Technology, Trondheim, Norway

**Keywords:** Virtual worlds, Virtual reality, Avatars, Digital twins, Business value

## Abstract

Metaverses refer to immersive virtual worlds in which people, places, and things of the physical world are represented by their digital representations. The wide adoption of metaverses is expected to widely disrupt the way we interact in the virtual world by elevating our online interactive experiences and bringing a plethora of implications for businesses. Following a structured literature review of related research published in the last decade, we shed light on our current understanding of metaverses and reflect on the potentially transformative value of metaverses for businesses in the near future. We draw on an established research framework to organize the insights of existing literature across different levels of analysis and activities’ purpose. Through this analysis, we reveal eight propositions on the changes brought by the use of metaverses and identify a number of open questions which could serve as future research avenues.

## Introduction

The term *metaverse* was initially coined by the science fiction writer Neal Stephenson in his 1992 book *Snow Crash* to refer to a computer-generated universe (Stephenson, 1992) – a massive collective virtual environment that simulates the physical world, where users can get together to play games, socialize, and work. In this paper, we define metaverse as an immersive virtual world in which people, places, and things of the physical world are represented by their digital representations (e.g., people are represented by avatars) and are able to meet, communicate, interact, and collaborate. The notion of a three-dimensional virtual world has existed since tools such as OpenSimulator, Second Life, and VR head-mounted displays (e.g., Oculus Rift) appeared in the market. The virtual world is defined as a reality-inspired, digital, multimedia, three-dimensional, online environment where users can interact through the use of avatars (Chandra & Leenders, 2012). Virtual worlds include certain characteristics (Badilla Quintana & Fernández, 2015). They have a three-dimensional format (i.e., the experience is more immersive than static images); they involve an active user role through the avatar and a collaborative relationship with other users who exist through their avatars in the specific virtual environment.

The maturity of technologies, including extended reality, human–computer interaction, artificial intelligence, blockchain, computer vision, edge and cloud computing, and mobile networks, is currently driving the wider use of metaverses (Lee et al., 2021). Beyond its technical backbone, the metaverse encapsulates digital twins, immersive user experience, content creation interfaces, user-generated consent, and economics (Duan et al., 2021). Alongside technological maturity, the social distancing measures applied during the pandemic led to increased use of virtual tools and the wider use of mobile technologies, thus triggering the drivers of metaverses’ adoption. For example, Decentraland^1^ is a 3D virtual platform that goes beyond interaction with users, as they can create, explore, and purchase virtual property through it, supported by non-fungible tokens (NFTs). 3D avatar-based virtual and augmented reality tools have been available for more than a decade. However, recent announcements by tech giants regarding enhancements of online social media and communication tools through the use of immersive virtual environments will likely radically increase the use of metaverses in the near future (Meta, 2021; Roach, 2021). As the use of metaverses brings new capabilities (e.g., more collaborative meetings via mixed reality) and challenges to businesses, it is likely to inspire changes in business strategies, operations, policies, and organizational structure.

Existing literature has focused on reviewing the technical aspects of the technologies related to metaverses. Lee et al. (2021) review and examine the latest metaverse developments on the state-of-the-art technologies that serve as metaverse enablers and reflect on the user-centric factors that enable metaverse ecosystems. Xi et al. (2022) focus on metaverses as a form of augmented reality (AR) and virtual reality (VR) and reflect on whether its use poses an increase or decrease in the difficulties of carrying out everyday tasks. Earlier this decade, IS scholars also focused on reviewing the existing literature on tools and technologies related to the metaverse technologies and their impact on specific application domains. However, this pool of research has used the term “metaverse” to refer to tools such as Second Life, OpenSimulator, head-mounted displays, etc. For example, Muller Queiroz et al. (2018) provide a review of virtual reality technologies in e-learning. Giannakos et al. (2021) conduct a review of empirical studies on organizational learning and exhibit how research in these areas has used metaverse-related technologies. Radianti et al. (2020) conduct a systematic review of how researchers have applied immersive VR head-mounted displays in higher education. Other scholars have focused on reviewing existing literature on 3D virtual worlds in collaboration and their impact on businesses (Bououd et al., 2013). Research on metaverses in the past decade has evolved along with the technology. However, we still have a limited understanding of how the use of metaverses can contribute to business value.

Creating value through the application of emerging technologies involves dealing with various social, environmental, and economic challenges that can impact various stakeholders in society (Grover et al., 2018; Mikalef et al., 2020; Pappas et al., 2018). Focusing on the firm level to examine the business value of information technology enables researchers and practitioners to reflect on the economic impacts of adopting information technology and its manifestations (Kohli & Grover, 2008). Demonstrating the potential value of investing in technology is fundamental to the contribution of the IS discipline (Agarwal & Lucas, 2005) while considering the role of businesses in creating shared value (Porter & Kramer, 2019). Scholars within IS research deal with the interrelations among data and data actors, technologies, and information within their social contexts (Pappas et al., 2018; Struijk et al., 2022). Thus, in considering how metaverses are transforming the relationship between the digital and physical worlds, it is important to examine different levels of analysis and activities to better understand how stakeholders interact in metaverses. However, despite the potential of metaverses to bring the next “digital big bang” (Lee et al., 2021), IS literature currently lacks a systematic review that reflects our current knowledge of the business value potential of metaverses. This gap signals a scarcity of sufficient information sources and leaves researchers and practitioners in uncharted territories.

To address this issue, we need to learn from the past and identify how research has examined the use of metaverses in the past decade. Thus, it is necessary to map existing IS research on the intersection of metaverses and business value to extract useful theoretical and practical implications and identify future research avenues. To this end, we perform a systematic literature review of existing research on the use of metaverses and offer guidelines and propositions for how the use of metaverses can drive business value.

More specifically, this paper is framed by the following research questions:

- RQ1: What is the status of research on the use of metaverses to create business value?
- RQ2: How can the use of metaverses be leveraged to enhance business value?

To structure our knowledge of the existing research landscape and identify emerging areas for future research, we draw on a framework that has been successfully applied in the context of social media and business transformation (Aral et al., 2013). This framework helps us organize existing research on the use of metaverses and business value. The contribution of this paper is two-fold. We contribute by mapping existing research on the use of metaverses and their impact on businesses and society and shed light on how existing research on metaverses has evolved over the years. We provide propositions for using metaverses to enhance business value, followed by a research framework with prospective paradigmatic research questions.

This paper holds theoretical and practical contributions. We formulate propositions and research questions that can assist researchers in investigating new business models to respond to the rapid changes in the tech arena or designing experiments to reflect on customer experiences in the metaverse. Additionally, the paper is expected to stimulate IS research on this topic. Although research on metaverses from a technical perspective has been conducted recently, its potential wide deployment urges the need for further research in light of its social, contextual, and management perspectives. A systematic literature review on the topic is expected to raise the interest of the researchers. Furthermore, metaverse promises a notable amount of business value to enterprises as it progresses. Thus, this paper offers practical guidance to organizational IT executives interested in understanding the potential value that the metaverse can bring to businesses and serve as a reference point for the changes businesses need to implement in the light of the metaverse era.

The rest of this paper is structured as follows. In Sect. 2, we describe the methodological approach followed for conducting a systematic literature review framed by the research questions of this paper. Next, in Sect. 3, we present the framework employed for analyzing the publications identified. In Sect. 4, we analyze the outcome of our literature review regarding the framework presented in Sect. 3. In Sect. 5, we reflect on the analysis of the review of existing literature to derive propositions regarding the use of metaverses and outline avenues for future research on this topic. Finally, Sect. 6 summarizes the key outcomes of this work and its implications.

## Methodology

### A structured Literature Review Approach

This paper aims to provide a framework for analyzing current research on metaverses and business value to guide future researchers in advancing research on this topic. To this end, we have identified and reviewed existing research on this topic in a structured approach. Then, through a guided content analytical approach, we have analyzed and consolidated information from the literature collected.

Aiming to review existing research on metaverses, we adopt a systematic literature review (SLR) approach to identify, filter, analyze, and interpret existing literature on this timely research topic (Kitchenham & Charters, 2007; Webster & Watson, 2002). Reflecting on the research questions framing this paper, we developed a structured process for identifying papers that could be included in our analysis. The SLR approach of Kitchenham and Charters (2007) is a popular systematic literature review method frequently employed in IS research (e.g., Alzoubi et al., 2016; Giannakos et al., 2021). In our SLR, while scoping the literature, we focused on extracting a comprehensive overview of relevant papers while remaining open to excluding papers on related topics that were not relevant to our research question (Pare et al., 2015). More specifically, our process encapsulated the definition of sources of the research scan, the means to retrieve the papers identified, and the criteria for the inclusion and exclusion of papers depending on their relevance to our research questions (Kitchenham & Charters, 2007). To ensure that important papers were not excluded from our review, we also followed Webster and Watson’s (2002) forward–backward search to identify additional potential articles that might not have been identified. This subsection describes the article collection, selection, filtering, and content analysis steps followed for this paper.

#### Article Collection

A comprehensive search approach was employed, aiming for a high-quality review of existing literature on the topic. Our article collection process included searching for conference and journal papers in databases that cover the major venues of IS publications, the AIS Senior Scholars’ Basket of Journals (basket of 8), AIS Electronic Library, Science Direct, and ACM. We used the search engine tools, utilizing the keyword “metaverse” and selected papers published in the last decade (2012–2022), as we noticed that significant advances had been made in this area in the last ten years. The search process uncovered 397 papers. Given that the focus of this paper is on consolidating scientific knowledge arising from scientific papers on the topic, our process excluded publications such as press releases, media, white papers, book chapters, communications, and encyclopedias. This step led to the identification of 183 papers.

#### Article Selection and Shortlisting

The selection phase is important, as it determines the overall validity of the literature review. Thus, defining inclusion and exclusion criteria was important. After the initial paper identification phase, we examined the tiles and abstracts of all papers that resulted from the search results. We followed Dybå and Dingsøyr (2008) to cover the three main issues to examine when evaluating the quality of shortlisted papers: rigor, credibility, and relevance. These criteria are listed in Table 1. We emphasized the relevance criteria to ensure that the paper focused on the application, use, design, impact, benefits, and challenges associated with the use of metaverse-related technologies for individuals, society, organizations, and industries. Similar criteria have been used in recent literature reviews in the IS field (Giannakos et al., 2021; Alzoubi et al., 2016; Gall & Pigni, 2021).

**Table 1:** Criteria employed for evaluating the quality of shortlisted papers

The study clearly addresses a metaverse-related research problem
The aim of the research is clearly described
The context in which the study was conducted is clearly described
The research approach employed is adequate for the research aim of the paper
The research methods employed are well described and sufficiently justified
The data analysis of the paper is rigorous
The findings are sufficiently explained
The study contributes to the relevant body of literature

**Fig. 1:**
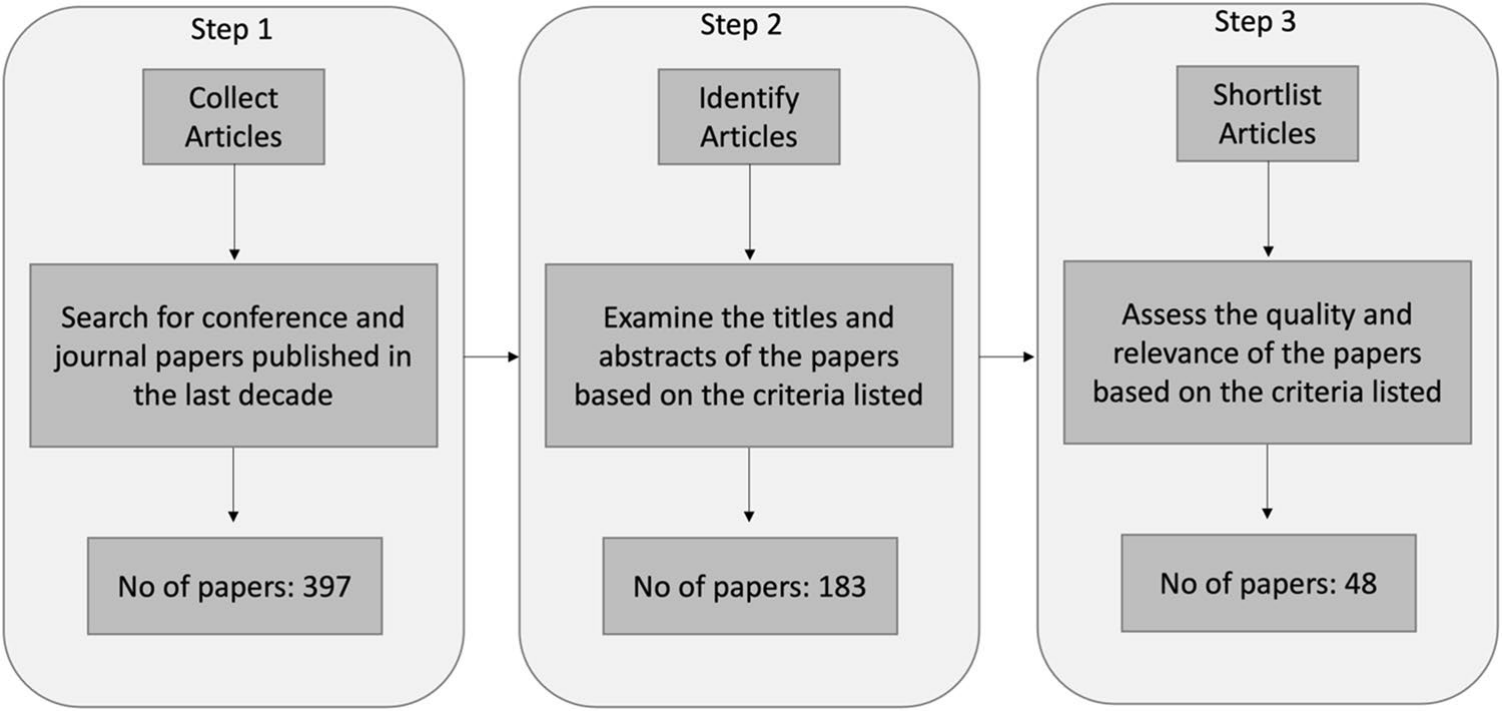
Stages of paper selection process

The criteria were mainly applied in Steps 2 and 3 of our paper selection and shortlisting process illustrated in Fig. 1. In Step 2, papers that were not relevant to the focus area based on the abstract were excluded, and in a few cases where the databases retrieved duplicates, papers were counted only once. In Step 3, we reviewed all shortlisted papers arising from Step 2. Papers that aligned with the criteria above were included in our review. We then followed the forward–backward approach of Webster and Watson (2002) to look into the citations of the articles identified to that point and search and identify articles citing the key articles identified in the previous steps. We looked for articles including the keyword “metaverse” in their title or abstract. Considering that our earlier search approach included a broad keyword (“metaverse”), the forward–backward search did not reveal additional articles. This approach also assisted us in verifying the article identification approach. This effort led to a final list of 48 papers. We note that reflecting on the criteria listed in Table 1, we were able to follow a consistent process across all databases and papers identified. The papers retrieved were also discussed among all authors, minimizing the risk of criteria subjectivity.

After finalizing the list of papers to be reviewed, a qualitative content analysis was followed. Figure 1 illustrates the different steps followed in this process, as well as the number of papers that emerged in each step of this process. This process led to a collection of a broad list of research papers from different disciplines, which provided insights for our content analysis.

#### Content Analysis

Aiming to develop a framework for organizing advancements relevant to metaverses, we followed Morris’ five-step process for directed content analysis (Morris, 1994). We adopted Aral et al.’s (2013) predefined categories and descriptions (cf. Sub-Sect. 2.2.). Reflecting on the five-step process of Morris (1994), for each paper, we began by determining the unit of analysis. We then categorized the papers with respect to the academic discipline and research method employed. We then employed the established framework structure by Aral et al. (2013) and applied it to collect and organize papers. The framework was then revised to better fit the specifics of the metaverse. Following this approach, we analyzed the final list of research papers following the Aral et al. (2013) framework. Divergences in the paper clustering were discussed among the authors to reach a final consensus.

### Background: An Organizing Framework for Metaverse Research

In this paper, we adopt the framework proposed by Aral et al. (2013) for reviewing the social media literature. Since then, other IS scholars built on this framework to review research in other IS domains, e.g., blockchain research in Risius and Spohrer (2017). Along the same lines, earlier versions of the metaverse (e.g., Second Life) have been compared with social media regarding the business opportunities they bring (Sharma et al., 2013). In their proposed framework, Aral et al. (2013) acknowledge the framework’s intention to guide research in social media and digital transformation. As the social media research area holds a plethora of similarities with metaverses (e.g., virtual profiles, communication, user interaction), we consider this framework a viable starting point for reviewing existing research on this topic. Aral et al. (2013) identify four thematic research areas that span three (overlapping) units of analysis. We employ these four thematic areas to describe and organize existing literature on metaverse-related technologies for business value:

1. Design and features: how users and organizations use specific features; how platforms, organizations, and governments design, implement, standardize, and regulate these features to foster or control the use of these technologies
2. Strategy and tactics: how users, organizations, and governments may use metaverse-related tools and create strategies (e.g., product development, pricing) that meet their requirements or goals
3. Management and organization: how users, platforms, organizations, and governments structure, manage, and provide the processes, resources, and infrastructure needed for the development, deployment, use, and interaction with metaverses to achieve their goals
4. Measurement and value: how users, platforms, organizations, and governments create, measure, or allocate the value generated by the use of metaverses.

Reflecting on Aral et al. (2013), their framework identifies four thematic areas of research and distinguishes three levels of analysis that can be employed to reflect on existing research from a specific viewpoint. The aim of this paper is to examine existing literature on metaverses and its applications with regard to business value. Thus, we apply this framework in a new context and adapt its thematic areas across the same three analysis levels to emphasize businesses and organizations while reflecting on the role of society or individuals due to their potential to impact business value creation from the customer’s perspective.

## Findings

### Overview of Structured Literature Review Results

**Fig. 2:**
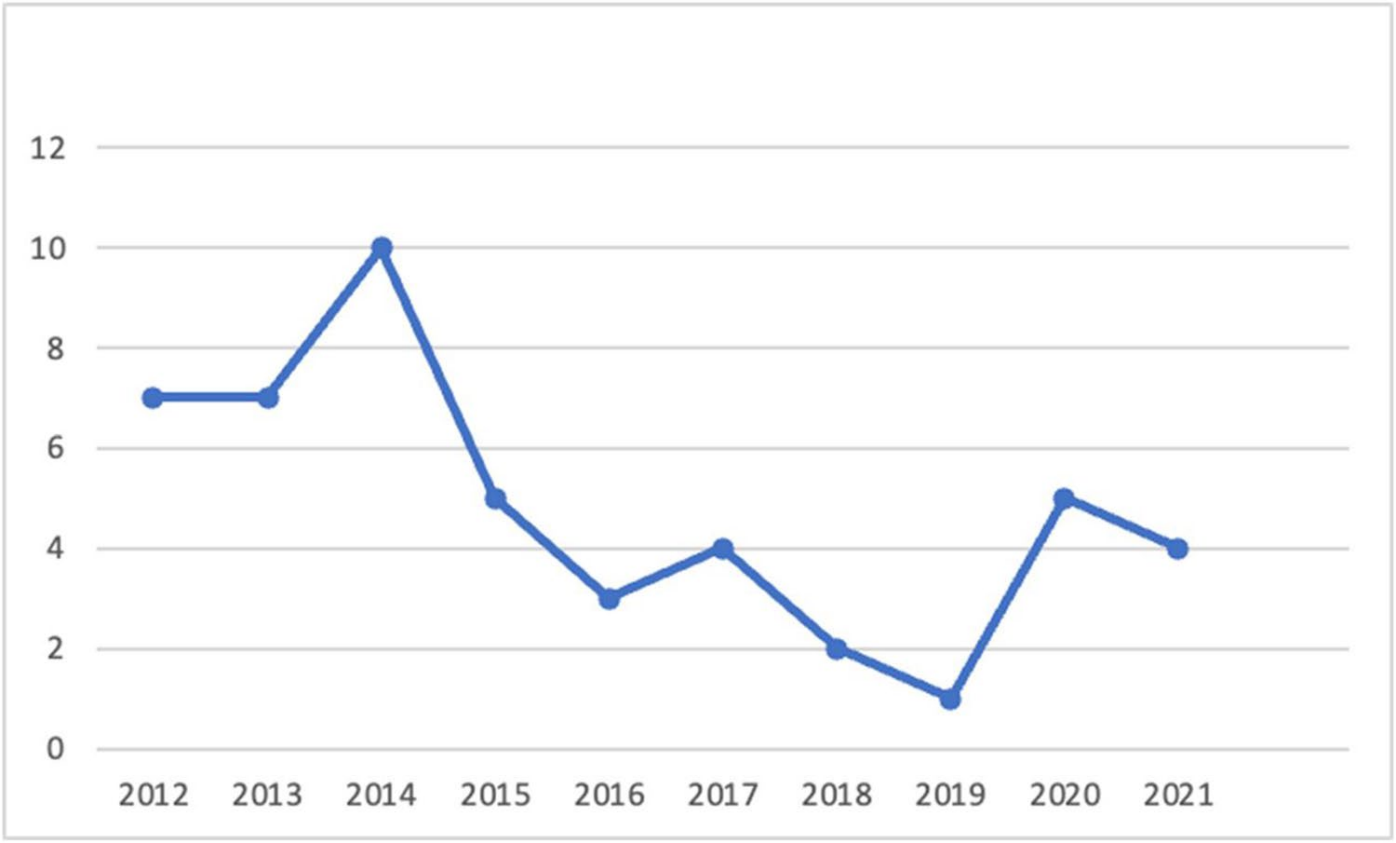
Number of papers on metaverses per year

In this paper, we reflect on existing literature to understand the business value of metaverses. In particular, 48 academic papers were reviewed following the criteria described in Sect. 2. Figure 2 illustrates the number of papers identified for each year of the past decade. We note that 2014 recorded the highest number of publications identified throughout our review. Of the 48 papers reviewed, 32 were published in academic journals, whereas 16 were published as conference proceedings. In Table 2, we summarize the outlets in which the shortlisted papers were published. The majority of the papers shortlisted were published in computer science or information systems outlets, whereas a few papers were published in cross-disciplinary outlets (e.g., education and information systems, psychology and technology). Comparing the papers published in computer science and information systems, we note that computer science conference proceedings and journal papers mainly focused on business value regarding elevating user experience, human–computer interaction, and enhancing the technologies that facilitate experiences relevant to the metaverse. Along the same lines, conference proceedings and journal papers on information systems were more relevant to topics such as improving consumer behavior and elevating business strategy. We also note that our analysis compared journal and conference papers. However, conclusions were limited because journal papers offered richer and more extensive insights. This might also be associated with the fact that the number of conference proceedings identified is notably smaller than the number of journal papers. Appendix, includes information on all the papers included in our review, as well as information on the area each paper tackles.

**Table 2:** Papers shortlisted and outlets

Journal Outlet	No. of Papers
Procedia Computer Science^1^	5
Computers in Human Behaviour	5
Procedia Technology	3
International Journal of Information Management	2
Technovation	2
Computers & Education	2
Expert Systems with Applications	1
Energy Procedia	1
The Journal of Academic Librarianship	1
Signal Processing: Image Communication	1
Procedia CIRP	1
Neurocomputing	1
Information & Management	1
Australasian Marketing Journal	1
Journal of Retailing and Consumer Services	1
Computer Standards & Interfaces	1
Journal of the Association for Information Systems	1
Communications of the Association for Information Systems	1
Journal of Management Information Systems	1
Conference Papers per Outlet	
Conference	No. of Papers
Virtual Reality International Conference	4
European Conference on Information Systems	4
ACM/IEEE International Conference on Human–Robot Interaction	1
Nordic Conference on Human–Computer Interaction	1
ACM SIGGRAPH Symposium on Applied Perception	1
Annual Symposium on Computer–Human Interaction in Play	1
International Conference on Web Intelligence, Mining and Semantics	1
Americas Conference on Information Systems	1
Mediterranean Conference on Information Systems	1
Hawaii International Conference on System Sciences	1
Total Conference and Journal Papers:	48

^1^Listed as a journal on Science Direct, although its description refers to high quality conference proceedings

By selectively surveying the research work of the past decade and organizing its contributions using the framework introduced by Aral et al., (2013; cf. Sections 2 & 3), we aim to structure existing knowledge on this topic and identify emerging bodies of work to guide future research. Below, we present the papers we reviewed with respect to the framework structure.

### Design and Features

#### Users and Society

Students may use metaverses to enhance their learning experiences using avatars. For example, in Barry et al. (2015), virtual problem-based learning for college students in virtual environments has been applied using avatars. Elementary school students (fifth and sixth grade) have used similar tools for blended education modes in STEM education (Kanematsu et al., 2014). Bououd et al. (2013) reflect on experiential learning in virtual worlds through teacher and student avatar representations that facilitate a high degree of social interaction in the virtual world. In Choi and Kim (2017), tourists had the opportunity to be involved in meaningful online and on-site museum experiences as they gained virtual access to exhibition content using augmented and virtual reality. Simulations are also more realistic in the virtual world. For example, Barin et al. (2017) explore drone racing sports and drone crashes to better inform on the reasons leading to crashes in drone racing. Through their research, the authors provide insights into drone design, advanced user interface improvement, and better support of human cognition and perception for pilots in drone racing.

Employees in virtual teams can improve their collaboration experience using virtual worlds (Suzuki et al., 2020). Especially for organizational teams in different geographical locations, a virtual collaboration environment that facilitates different communication channels and enables the manipulation of digital artifacts can assist teams collaborating in different locations (Boughzala et al., 2012). Along the same lines, entrepreneurs and innovators may use virtual worlds as a means of collaboration. For example, Chandra and Leenders (2012) consider this an environment to share knowledge and resources to achieve user innovation in both virtual and real-life settings. Reflecting on innovation co-creation potential in the digital world, customers using tools such as Second Life can take an active role in the application of ideas, processes, products, and procedures that are new to the adopting organization (Riordan et al., 2012). Artists can also benefit from such technologies to simulate an online virtual environment and a real-life art exhibition (Carneiro de Sousa, 2014). Beyond professional artists, existing literature also provides evidence for students who worked collaboratively on design using virtual worlds. Represented by their avatars, the students could discuss and design eco-cars in the virtual world (Kanematsu et al., 2013). Research on designing avatar faces has also demonstrated the ability to convey to the observer the most relevant sensations in accordance with the context (Diego-Mas & Alcaide-Marzal, 2015).

### Intermediaries and Platforms

Virtual school and campus experiences can be provided in metaverses. Intermediaries and platforms offering learning material in the physical or the digital world can complement their offerings by providing learning experiences in virtual worlds. As a result, they can contribute to synchronizing and extending real-life teaching platforms (Kanematsu et al., 2014). Such experiences can also enhance student experiences and learning approaches at different educational levels. For example, virtual learning campus tours were made possible for university students (Crespo et al., 2013), and virtual worlds enabled students to share their emotions regarding problems they are invited to solve (e.g., blinking avatar; Barry et al., 2015). Companies developing infrastructure for educational purposes or even offering distance learning platforms for education purposes need to meet requirements for the implementation of future metaverses.

Technologies such as head-mounted displays are already available to museums aiming to enhance the content service experiences of their visitors (Choi & Kim, 2017). Tech companies may continue to explore technological solutions that can be installed in museums and other sites to elevate the experience. Similarly, tourists may be able to visit places virtually from the comfort of their homes. In this respect, technology providers may consider how to transfer sensed information (e.g., temperature, humidity, light intensity, gas intensity) from real-world to virtual-world objects. Existing research has already made steps in this direction, e.g., Kim et al. (2013) developed a system that uses a real-world mock-up equipped with a sensor set and a virtual space.

### Firms and Industries

Winkler et al. (2020) shed light on individual user immersion associated with the use of virtual worlds. In their analysis of immersion factors in virtual reality environments, they highlight physical and physiological as well as cognitive and affective factors, including translating actions from physical to virtual worlds, distracting aspects, losing sense of time, and affective involvement in the virtual reality world. These findings highlight potential negative psychological consequences of using a virtual environment. Drawing on the use of VR head-mounted displays (e.g., Oculus Rift), Kampling (2018) reveals that temporal dissociation is related to the cognitive absorption experienced when using such tools. Sebastien et al. (2018) introduce the IMMEX program, which is composed of different programs, each corresponding to a metaverse that simulates the university environment. These include outdoor campus, indoor building experience, and complete experience (interiors and exteriors of buildings can be visited). The authors experiment with various services in these metaverses, aiming to offer users alternatives for discovering and/or sharing information in a ludic way. Empirical feedback from young students regarding the features of these worlds is more encouraging than the feedback from senior individuals. Reflecting on the development of the digital identity of adolescents within virtual worlds, McKenna and Vodanovich (2016) stress that the prolonged use of virtual spaces (e.g., games) and the digital identity developed throughout this process hold a high impact on the individual’s personal identity development. The use of virtual spaces may help or hinder their personal identity development based on their personality type and other factors. Thus, it is important for technology intermediaries to consider these aspects when designing their own metaverses, especially if they target specific audiences, e.g., children and young adults. Such findings also signal the need for mental health policymakers to provide recommendations and training to minimize the negative psychological and behavioral consequences associated with the prolonged use of virtual environments.

### Strategy and Tactics

#### Users and Society

Suzuki et al. (2020) highlight that remote teams can collaborate in the virtual world such that team members simulate face-to-face team collaboration, including mutual communication. They suggest that through their avatars, team members can use text communication tools (e.g., chatting) to enable communication among multinational teams with members speaking different languages using an automatic language translation system (Suzuki et al., 2020). Employees in organizations may consider using such technologies to better support collaboration among geographically sparse multi-lingual teams. Similarly, the creative process of artists can be elevated through the use of virtual worlds, as this generates new creative opportunities for artists and gives them the potential to experience a shared creative experience of avatar design (Tang, 2021). This process gives them opportunities to use different strategies for shared creativity, which has implications for the strategy followed by artists when it comes to collective creation, i.e., the creative process in which participants act as one creative entity and distribute creativity. In the former, all artists hold equal participation, and based on the results of the experiment, it is difficult to achieve in large groups. In distributed creativity, artists can contribute to building on and extending an existing pool of avatars (Carneiro de Sousa, 2014).

Metaverse-related technologies have also enabled teachers to develop new teaching strategies and to ensure business continueity in the education sector during the COVID-19 pandemic. This was achieved through the shift to remote teaching strategies (Barry et al., 2021). Technologies such as Microsoft Teams and Zoom have been employed to promote synchronous and active student learning experiences during the pandemic. Finally, Bello-Orgaz and Camacho (2013) perform text-clustering techniques for text used in virtual worlds in a university context. Their research demonstrates that most conversations in virtual classrooms are associated with group work, whereas other conversations include welcoming conversations that occur when students enter the virtual world and instructions on using the tools included in virtual worlds.

#### Intermediaries and Platforms

A new role is arising for metaverse platforms to support introductory experiences of individuals and teams or as new forms of marketing campaigns. For example, in Tang (2021), the university library employed an AR treasure hunt game to assist the library orientation for first-year college students. This support towards introductory experiences has implications for gaming platforms that may act as intermediaries between individuals and organizations interested in providing an immersive virtual space experience for a group. Research on the determinants of friendships between users in the virtual world for users who are not aware of each other’s real identities highlights that avatars are in favor of initiating friendships with other avatars that have a different appearance (e.g., dressed differently from each other), with avatars that seem to be in a closer virtual location in the virtual world, and with avatars which tend to use fewer words for their communication (Chesney et al., 2014). Gaming companies serving organizations interested in employing virtual worlds to support a specific business goal (e.g., marketing companies designing metaverses to engage their customers) may reflect on such findings to formulate their virtual gaming strategies. Webster et al. (2017) focus on virtual reality as an opportunity for music composition in the context of a 3D scene in perspective/projection mode. Through their project, the authors aim to assist the user to feel wholly in the virtual space and experience the user/avatar fusion in the 3D space. Ylipulli et al. (2016) focus on combining a virtual 3D city and the real world (e.g., users interacting through mobile devices) into a hybrid urban space. They use a scenario to problematize certain questions on the interaction with hybrid worlds. Through their approach, Ylipulli et al. (2016) reflect on the perception of nearby and distant places in a hybrid city and conclude that hybrid cities may change people’s perception of a certain physical place. Municipalities and urban design consultants can potentially use VR to simulate citizens’ experiences when considering upcoming urban landscape changes. For example, Luo et al. (2022) use a VR-supported viewing experiment to simulate people sitting in pavilions in urban parks and derive conclusions from their experiences. Through their study, the authors evaluate the citizens’ preferences and the mental restoration potential of such pavilions and further explore their impact on well-being and quality of life, demonstrating that pavilions effectively promote mental restoration.

#### Firms and Industries

Several industries may consider a radical shift in their strategies regarding the metaverse. Industries may consider changes in their business models, such as the channels they use for communicating with and engaging their target audiences, the infrastructure employed, the resources needed as well as their revenue streams. For example, the museum industry may benefit from new exhibition strategies and explore new technologies (e.g., head-mounted displays) and make physical space arrangements (e.g., exhibition rooms) to accommodate the virtual-world experiences of their visitors. Along the same lines, in the arts industry, Amato et al. (2012) explore the impact of an interactive installation in an artistic context. The installation invited avatars and humans to co-participate in a simulated environment, demonstrating that this experience produced an intermediate world between the two worlds. The mirroring and dialogue of humans through technology enabled the participants to examine their own fundamental existential condition.

Education provides a need to enhance blended and e-learning strategies, as evidence from existing research highlights that metaverses can significantly contribute to raising the learning experience for students. For example, the use of such tools assisted students in the process of engineering design (Kanematsu et al., 2013), STEM (Kanematsu et al., 2014), problem-based learning (Barry et al., 2015), and others. Along the same lines, the metaverse has the potential to largely affect the media industry in several components of its business model. Mütterlein and Hess (2017) analyze different uses of virtual reality in this industry, including internal use (e.g., conferencing and collaborating) and external use, such as the production and distribution of VR content (e.g., videos and games). Regarding external use, the authors show that the impact of VR requires more technologies to create more interactive content, which brings implications for changes in the business models of the media industry.

### Management and Organization

#### Users and Society

Teachers and academics need to adjust their pedagogical methods and course syllabuses to better facilitate virtual or blended learning experiences. Drawing on the immersive virtual environments, Badilla Quintana and Fernández (2015) develop a pedagogical model to assist teaching staff. Compared to traditional tools employed for online teaching (e.g., Zoom, WebEx), virtual learning experiences enable teachers to better simulate physical classroom experiences. Thus, teachers are more empowered, as they can better understand students’ emotions in virtual problem-based learning environments (Barry et al., 2015). Thus, pedagogical methods and course syllabuses need to be enhanced to better facilitate and account for such enhanced learning experiences. Collaborative networks in the virtual setting can serve as a significant source of new knowledge and resources for team member innovation and entrepreneurship (Chandra & Leenders, 2012). In their experiments, Riordan et al. (2012) show that through Second Life, users can contribute to innovation and co-creation in the virtual world. Other research explores potential VR software improvements and the extent to which they could support the way work is contacted in geographically distributed teams in the area of product development. Given the benefits of virtual worlds for team collaboration, employees may consider enhancing their skill sets to smoothly adapt to creating 3D objects in the virtual environment (Balzerkiewitz & Stechert, 2020). Herbet (2014) reflects on interactions in a collaborative virtual environment where users are co-located in the same physical space such that they also have a physical presence during their virtual interaction. Through her experiment, she explores the perception of presence and concludes that it is increased if full-body avatar conditions are enabled. Thus, the form of the avatar is important and highlights that physical collocation is not important.

#### Intermediaries and Platforms

Intermediaries need to focus on retraining user groups. As we move towards virtual worlds of experiential learning, the role of the educator is changing to motivating and moderating student discussions rather than introducing students to content through presentation (Lattemann & Stieglitz, 2012). Thus, the HR departments of schools and other educational organizations responsible for lifelong teacher training need to offer training to teachers. In particular, they need to enhance their training portfolios with new didactical and technical skills that will enable teachers to “e-moderate” students and apply different teaching methods in the virtual worlds (Lattemann & Stieglitz, 2012). Organizational HR departments and professional training organizations must also enhance their training schemes to train employees to use collaboration tools in the virtual worlds and enable them to fully benefit from these tools. As highlighted by existing literature, new sharing and collaboration platforms, as proposed by Suzuki et al. (2020), elevate how remote organizations collaborate, leading to an enhanced metaverse experience in team collaboration. Through their analysis of the challenges associated with hybrid worlds, Ylipulli et al. (2016) note the need for regulating digital traces, similar to what is currently followed in social media spaces once an individual passes away. Young et al. (2015) focus on immaterial art stock to establish a protocol and method to preserve artwork generated in 3D digital spaces. The authors highlight the need for continuous maintenance and animation support for spaces that include such artwork as well as increasing the general public’s awareness and potential to discover such artwork in a formal setting and have the opportunity to meet the artwork creators.

#### Firms and Industries

The use of metaverses also imposes changes in the processes of many industries. For example, in the museum industry, the use of metaverses is changing how exhibits are organized to elevate the visitor’s experience (Choi & Kim, 2017). It also brings notable changes to the marketing industry. Augmented reality marketing (ARM) and related technologies can deliver valuable consumer experiences that differ from traditional marketing approaches (Chylinski et al., 2020). The work of Papagiannidis et al. (2014) demonstrates how simulation through virtual tools can serve as the foundation for developing a relationship between a user and the simulated product. Thus, firms may consider offering potential consumers the possibility to experience simulated products, which is valuable, especially if we consider the increasingly important role of “experience” in online marketing. Relationships in virtual environments are also relevant to trust development. Data arising through fMRI experiments (Riedl et al., 2014) show that when it comes to the trust of humans with avatars in virtual environments, people are better able to predict the trustworthiness of humans than of avatars, whereas the trustworthiness learning rate is similar with humans and with avatars. This brings additional implications to the field of e-commerce and the use of avatars as sales assistants.

Additionally, traditional exercises to improve emergency management can be better supported using virtual reality (Ardila et al., 2015). In particular, VR can support traditional exercises to enhance the capabilities of real-world applications. The authors propose a new architecture for a training system that interconnects the real and virtual worlds and facilitates real-time training (Ardila et al., 2015). In Vallance (2013), the author brings together students from different countries to collaborate in a 3D virtual world, aiming to better understand the challenges and suggest possible solutions relevant to disaster recovery (e.g., Fukushima). Along the same lines, the use of VR changes the processes in the education sector. To offer learning opportunities that include 3D virtual worlds, educational institutions need to adjust their processes and IT management resources to manage their own virtual-world servers, including relevant application development, server scalability, and cost management (Miller et al., 2012). Thus, for potential educational virtual worlds to be realized by educational organizations, the organizations need to facilitate the application design, support for resource creation, and system support for all students and staff using technologies relevant to metaverses. Through their research, authors highlight that beyond accommodating, schools will also need to address new technical difficulties such as failure to identify students behind avatars and chat situations when reading and writing occur at the same time (Badilla Quintana & Fernández, 2015).

### Measurement and Value

#### Users and Society

The use of metaverses brings value to users. This may include positive emotions and enhanced experiences. For example, students identified positive experiences associated with the use of virtual worlds. Regarding supplementing hands-on experiments with a virtual class through metaverses, (Kanematsu et al., 2014), students reported positive experiences with regard to the level of information received, highlighting that the virtual world allowed them to become more conformable. Further, the new generations of students are already familiar with technologies. Thus, it is to some extent expected that these technologies will be part of the e-learning and teaching process involving the use of virtual worlds and social networks. Indeed, existing literature demonstrates that such practices can support students in their learning journey (Badilla Quintana & Fernández, 2015). Through their five-year experiment in enhancing exploratory learning using virtual worlds, Miller et al. (2012) concluded that the virtual worlds technology enabled student presence in the virtual environment through the proxy of avatars. In particular, they highlight that the simulations of different learning experiences (e.g., archeological dig, teaching space for a management course, laboratory for wireless networking, and human–computer interaction) demonstrate positive student experiences through which students have been involved in valuable educational activities that would have been impossible without the use of technologies that supported their representation in the virtual world (Miller et al., 2012). In his research on adult language learners, Chen (2016) sheds light on the use of Second Life (SL) as part of the language learning experience as well as the effects of task-based instruction on learning a language using SL. The author demonstrates that SL has been a viable learning environment in their study due to its “conspicuous features, immersive and virtual reality, sense of tele- and co-presence” (Chen, 2016). In particular, it shows that SL provides the language learners with visual and linguistic support and thus contributes to language teaching and learning, whereas using tasks that draw upon SL features have the potential to facilitate world knowledge and cultural knowledge of the learners as they may simulate real-life scenarios (Chen, 2016). The user-experienced foreign language anxiety in the virtual world is likely to be reduced compared to the real world (Grant et al., 2014).

Beyond e-learning, avatars and user identification in the virtual worlds are important for constructing attractive virtual worlds. In other words, how users build trust in a virtual community is an important element of their satisfaction in the virtual world, can enhance their efficacy and trust, and thus sustain the use of virtual services (Kim et al., 2012). Drawing on Griol et al. (2019), the selection of system response needs to be adapted and consider the information that has been stored in the user’s profile as well as the emotional content arising from the user’s utterances. Luse et al. (2013) examine user reactions in the virtual worlds, such as users’ attitudes in Second Life (before exposure, during the information session, and after use of the environment). Their study shows that self-efficacy increases over time, whereas user acceptance decreases in a highly correlated pattern. They also shed light on the underlying causes related to user acceptance, including user intentions. In Papagiannidis et al. (2014), the authors focus on user experience and engagement in virtual test-driving and study their determinants. Their study suggests that hedonic experience is related to a higher level of user engagement and enjoyment, which also positively influences user satisfaction associated with driving the simulated car. The emotions experienced by users are likely related to the motives of the users to participate in metaverse-mediated platforms. Motivations are based on roles as “Role-Players,” “Relationship Seekers,” “Manipulators,” “Achievement Seekers,” “Friendship Seekers,” “Uninvolved,” and “Escapists” (Hassouneh & Brengman, 2014). These motives may differ based on demographic data associated with age and gender.

#### Intermediaries and Platforms

The use of metaverses brings indirect value to intermediaries interested in the environment. For example, governments and environmental associations interested in reducing transport emissions may benefit from the wide use of virtual worlds. The use of metaverses holds the potential to reduce emissions by virtualizing or eliminating some human activities that are harmful to the environment. In particular, through the wide use of virtual worlds, we can minimize humans’ climate impact while ensuring the quality of human life, as this can be achieved through the redesign of human work-related processes such that they reduce commuting needs (Trkman & Černe, 2021). Government and environmental associations may build on these opportunities and develop policies that encourage the adoption of metaverses in the work context to support redefining human work-related processes (Trkman & Černe, 2021). Platform owners may expand their value-generation streams while real-world business revenue can arise through virtual-world properties. In this respect, value can be generated through ownership ambiguity regarding the separation of content and platform ownership (Zhou et al., 2018). As noted by Zhou et al. (2018), the current virtual world business model favors the platform’s ownership rights compared to other competing ownership interests. At the same time, this poses challenges for virtual user innovators due to competing ownership interests.

#### Firms and Industries

Several industries may benefit from the use of metaverses. E-retailers may gain value through the sales of virtual products, as users may identify new consumption needs in virtual worlds. Existing research shows that virtual consumption enables a diversity of goals beyond the previously identified experiential goals as users experience transitions between their real-world and virtual identities, which impacts their virtual consumption (Jung & Pawlowski, 2014). Education institutions are able to offer enhanced learning experiences that elevate the effectiveness of blended or virtual learning approaches (e.g., Barry et al., 2015). In the archeology sector, virtual archaeology may also allow human interaction with the archeological environment, which is an important element of research for historians and archaeologists (Sequeira et al., 2014). In collaboration with technical teams, archaeology projects may draw on virtual tools that will enable them to simulate human interaction (e.g., model crowds) to scientifically validate their results. This may also bring indirect implications to the museum industry as it will elevate visitors’ experience with exhibitions supported by virtual-world simulations of human interactions and crowd modeling approaches (Sequeira et al., 2014). The use of metaverses brings value to businesses in different sectors. Researchers may consider how firms could best measure the outcomes of using metaverses.

Overall, this analysis sheds light on the intersection between metaverses and business transformation. In particular, through our analysis, we have dismantled the transformational impact regarding three levels of analysis: users and society, intermediaries and platforms, and firms and industries. The Appendix lists all primary studies included in the systematic literature review.

## Discussion

Our analysis has enabled us to reflect on existing research on metaverses and business value of the last decade. Building on the framework introduced by Aral et al. (2013), we have organized existing knowledge to examine its relevance to business value. Our approach allowed us to reflect on existing research to better illustrate its contribution from the user, intermediary and platform and firm and industry perspectives. Along the same lines, we have clustered prior research on four categories based on their relevance, including design and features, strategy and tactics, management and organization, and measurement and value. To this end, our work contributes to information systems literature by providing a structured review of existing literature featuring metaverses and business value. We foresee that this can assist future researchers in understanding existing knowledge on metaverses and identifying avenues for further research. In this section, we build on the results presented in Sect. 3 and further elaborate on implications for the current and future status of research on metaverses and business value. This approach leads to eight propositions that highlight the advancements expected using metaverses and connects our previous knowledge of virtual worlds to future research avenues that contribute to unlocking metaverses’ full potential for businesses.

### Propositions

The use of metaverses brings a new spectrum of interaction capabilities to the virtual world. From virtual problem-solving in schools to social interaction between students and teachers, the metaverse elevates the potential of the education sector to simulate and enhance learning experiences. Beyond education, as discussed in the previous section, virtual environments are also helpful for facilitating discussions, enhancing collaboration, and elevating innovation and co-creation potential. The use of avatars and the possibility to design avatars that imitate the appearance of the customer in the real world provide opportunities to create digital identities that simulate the real-world identities of users. Additionally, the ability to communicate sensations or emotions in accordance with the context via avatars elevates the potential of the metaverse to convey physical-world consumer experiences in the virtual world, thus elevating the overall customer experience. For example, in Choi and Kim (2017), consumers had the opportunity to participate in combined virtual and physical museum experiences. This may also help consumers visualize products that are not available or have not yet been created.

- ***Proposition 1:**** The use of metaverses introduces new ways of business-to-consumer interaction that enable the simulation of the physical world in the virtual world.*

To make the most out of their interaction in the metaverse, users need to enhance their skills and competences to participate in the metaverse. As revealed by existing literature in education, students can participate in new experiences, and teachers are able to use new, innovative learning methods. For example, in Barry et al. (2015), teachers feel empowered when they are able to understand students’ emotions in virtual environments. Along the same lines, employees of organizations can elevate their collaboration experiences, and create, distribute, and sell products in the virtual environment. Museum visitors need to learn how to use the relevant equipment, and so on. Similarly, customers may need to be trained to make the most out of their metaverse interaction with the business. To improve the transition to the metaverse and ensure the smooth and effective use of metaverses, users will need to develop new skills and adjust their older practices to the functionalities of virtual worlds.

- ***Proposition 2:**** The effective use of metaverses requires skills enhancement for employees and customers.*

Existing research demonstrates that the use of virtual worlds can have an emotional impact on individuals. For example, research has recorded hedonic experiences and high levels of enjoyment in simulated virtual environments (e.g., Papagiannidis et al., 2014). Trust in virtual worlds also evolves alongside user acceptance in virtual worlds, whereas emotions associated with participation in virtual worlds are likely to affect users’ overall motives in metaverse-mediated platforms. Alongside positive experiences, the use of metaverses can also have a negative impact, including negative psychological consequences and temporal disorientation. Thus, the emotions arising from the use of metaverses may change the overall well-being and quality of life of employees and customers participating in the metaverse.

- ***Proposition 3:**** Participation in metaverses impacts the emotional states of employees and customers.*

Participation in metaverses requires each entity to create and maintain an identity. This brings a plethora of changes to how digital identities are managed and pursue content, distribution, and sharing. Thus, new opportunities arise for intermediaries to serve as identity managers and content creators for different user types, including individuals and businesses (e.g., McKenna & Vodanovich, 2016). Along the same lines, identity creation in virtual worlds alters traditional approaches to authentication or introduces alternative approaches. For example, blockchain may directly serve authenticators without needing state-issued identity documentation; this may lead to disintermediation, as the state intermediary will no longer be required for identity confirmation. Identity may also be challenged at the perceptual level, as existing research indicates that prolonged exposure in virtual spaces may impact an individual’s personality development. This raises further implications for psychologists supporting individuals in handling their co-existence in the real world and the metaverse.

- ***Proposition 4:**** The use of metaverses imposes the creation and management of business and customer digital identities in the virtual world.*

Existing literature demonstrates the use of virtual worlds by individuals of different ages, including children (e.g., Bououd et al., 2013; Kanematsu et al., 2014). Among others, it demonstrates the benefits virtual worlds can bring to individuals, such as enhancing learning experiences, boosting innovation potential, and increasing collaboration, as well as the benefits for organizations (cf. proposition 8). However, alongside these benefits, using such environments requires generating and using avatar representations for users, whereas organizations can be represented through their employees’ avatars or through the creation of their own representations in the metaverse. The use of metaverses requires the accurate representation of physical world entities in the virtual world. This may expose individuals or organizations to security and privacy risks associated with the use of metaverses (e.g., risk of avatar theft). To address the new security and privacy challenges, regulations on privacy and security need to be adjusted or implemented to minimize these risks.

- ***Proposition 5:**** The use of metaverses imposes the need for regulation on business participation in the metaverse.*

Interaction in the virtual worlds requires the use of certain hardware and infrastructure. Some of these technologies identified by existing literature are head-mounted displays (Radianti et al., 2020), sense-transferring equipment (Kim et al., 2013), virtual reality labs (Winkler et al., 2020), servers, and other technologies that have been used to enable simulation of physical world experiences in the virtual world or even the synchronization and extension of real-life into the virtual world. Organizations aiming to enhance their employee or customer experiences using metaverses need to consider the technical requirements for such offerings and consider offering the necessary equipment and infrastructure for their target audiences (e.g., their customers).

- ***Proposition 6:**** The use of metaverses requires businesses to fulfill certain technical requirements.*

The presence of organizations in the virtual worlds brings changes to the way organizations operate. For example, in the education sector, different teaching processes are followed in the virtual worlds to facilitate student collaboration and fulfill teaching aims (Kanematsu et al., 2013), pointing towards how AI-based learning systems can evolve (Kabudi et al., 2021). The COVID-19 pandemic shows how important it is to redefine and rethink how learning is designed and conducted (Pappas & Giannakos, 2021). Along the same lines, routines for the employees of educational organizations change (e.g., setting up museum exhibitions that will accommodate virtual-world experiences). Regardless of the industry, participation in metaverses imposes changes in how different tasks are fulfilled or even generates new tasks for certain departments (e.g., the IT department needs to manage resources for operating the organization in the metaverse). Thus, organizations need to consider adjusting their operations to accommodate the tasks associated with their participation in metaverses.

- ***Proposition 7:**** Participation in metaverses requires changes in the routines and processes of organizations.*

Although participation in metaverses may partly change an organization’s operations, it also brings value to the organization by providing new business opportunities. Metaverses provide businesses with new means for developing and sustaining customer relationships as they release a new spectrum of valuable customer experiences. This may include additional marketing approaches (Chylinski et al., 2020) or opportunities for developing new tools or processes to support employee-driven digital innovations (Opland et al., 2022). Beyond new ways of advertising products (e.g., augmented reality marketing), there are additional avenues for reaching customers and new selling channels (Dwivedi et al., 2021). Along the same lines, firms need to develop new mechanisms and metrics to measure the impact of their participation in the metaverse and take corrective action where needed. Furthermore, businesses can provide new offerings in the metaverse in the form of virtual products and services which can be used in or supported by virtual environments (e.g., emergency management).

- ***Proposition 8:**** The use of metaverses releases a new range of business opportunities and thus adds value to organizations.*

### Future Research Avenues

Research on metaverses is ongoing as their potential to be integrated in multiple ways in our lives is high, raising the need for ways to design and develop metaverses to unlock their potential to contribute to the well-being of humanity. To do this, we argue that metaverses should have inclusive design. Design for inclusive metaverses starts by providing access to a diverse group of users and then ensuring their engaged participation, eventually leading to empowered success (Patrick & Hollenbeck, 2021).

In this section, we synthesized the results of our structured literature review across the three levels of analysis. This process led to the identification of eight propositions highlighting the changes expected by the use of metaverses. Following up on the propositions identified also led to the identification of prospective research questions on future research avenues related to metaverses. Reflecting on users and society, Proposition 1 invites future research to specify the design features that will enable the accurate simulation of the physical world in metaverses and how these could elevate the experiences of the customers in a metaverse environment. Proposition 2 stretches the need for further research on skills enhancement for employees and customers and how users could be retrained to make the most of their interaction in metaverses. Proposition 3 highlights new research avenues to study the impact of the use of metaverses on the emotional and mental states of employees and customers. Along the same lines, for intermediaries and platforms, Proposition 3 poses additional research questions for the role of platform providers in supporting identity creation, handling identity ownership, and managing customers’ identities in metaverses. Proposition 4 reflects the need for managing business and customer identities in the metaverse. Proposition 5 inspires new research avenues relevant to the new opportunities for regulating the use of metaverses by businesses aiming to minimize the risks of metaverses’ use. This may also pose opportunities for security intermediaries offering such services for businesses in metaverses. Regarding firms and industries, Proposition 6 highlights the need to research emerging technologies and identify how they could best serve firms participating in or developing metaverses. Proposition 7 calls for future researchers to explore the emerging changes in the processes and routines of businesses to accommodate the use of metaverses. Finally, Proposition 8 introduces future research avenues for new business models imposed by the use of metaverses as well as changes in business strategies and approaches for measuring value added by the use of metaverses for businesses. Table 3 summarizes the research questions identified for each level of analysis and activity area. We further illustrate the relevance between the framework and the propositions (indicated with the letter “P” in the table) as illustrated below.

**Table 3:** Multidisciplinary metaverse research framework with prospective paradigmatic research questions

Level of analysis	Activities			
	Design and features	Strategy and tactics	Measurement and value	Management and organization
Users	P1: *The use of metaverses introduces new ways of business-to-consumer interaction that enable the simulation of the physical world in the virtual world*	P3: *Participation in metaverses impacts the emotional states of employees and customers*		P2: *The effective use of metaverses requires skills enhancement for employees and customers*
	RQs: How do users interact with metaverse features? How do metaverse features serve the objectives of users? How can sensed information be transferred from real-world to virtual-world objects?	RQs: How do users make the most out of their use of metaverses? What strategies could elevate user experience in virtual environments?	RQs: What is the emotional and behavioral impact of metaverses on users? What are the factors shaping positive user experiences?	RQs: How should users adjust their routines to better facilitate the use of metaverses? What skills do users need to develop to benefit from the use of metaverses?
Intermediaries and platforms	P6: *The use of metaverses requires businesses to fulfill certain technical requirements*	P5: *The use of metaverses imposes the need for regulation on the participation of businesses in the metaverse*		P4: *The use of metaverses imposes the creation and management of the business and customer’s digital identities in the virtual world*
	RQs: How can metaverse platform providers and intermediaries support the appropriate creation of user and business identities in the virtual world?	RQs: What are the new market opportunities for security platforms and intermediaries? What issues should metaverse include?	RQs: What is the impact of ownership ambiguity in virtual worlds for platform providers? What is the environmental impact of using metaverses, and how can negative consequences be prevented by regulations?	RQ: How should platform providers manage identities?
Firms and industries	P6: *The use of metaverses requires businesses to fulfill certain technical requirements*	P8: *The use of metaverses releases a new range of business opportunities and thus adds value to organizations*		P7: *Participation in metaverses requires changes in the routines and processes of organizations*
	RQs: What technologies should firms consider when participating in or developing metaverses in order to best serve their business goals?	RQs: How should firms transform their business strategies to use metaverses? What are the implications of metaverses for current business models?	RQs: How do metaverses add value to firms in different industries? How can firms measure the business value of using metaverses?	RQ: How should firms adjust their business routines and processes to accommodate the use of metaverses?

## Conclusions

### Theoretical and Practical Implications

This paper provides an overview of existing research on metaverses. Our analysis shows that the existing research has emphasized the use of metaverses in specific sectors, including education, tourism, archeology, art, and others. The results demonstrate the applicability and importance of metaverses for a number of areas and showcase that the metaverse research landscape is rich and varied. Drawing on the framework of Aral et al. (2013), we were able to elaborate on existing literature on metaverses with regard to different levels of analysis and activities. As a result, this analysis can serve as a reference point for researchers interested in further exploring metaverses and business value. Additionally, it can assist researchers in understanding how, when and why metaverses creates value for businesses (Kohli & Grover, 2008) based on different levels of analysis and activities. We contribute to the ongoing discussion in IS that seeks to better understand the interrelations among data, information technologies, and different stakeholders within their social contexts (Agarwal & Lucas, 2005; Pappas et al., 2018; Struijk et al., 2022) and how they impact value creation for businesses.

Considering how metaverses can blur the lines between the digital and physical world and transform our daily lives, it is important to examine different levels of analysis and activities to capture the complex interactions among the various stakeholders and the technologies they use. Metaverse is a socio-technical phenomenon which is interleated with users as it brings significant changes to their everyday interaction with the virtual and physical enviroments. Similarly it is interrelated with intermediaries and firms as it alters they way they participate in the digital and physical worlds in order to reach and engage with their customers. Recent literature (and practice) has largely focused on reviewing the technical aspects of the technologies related to metaverses (Lee et al., 2021; Xi et al., 2022). However, in IS research it is critical to consider the sociotechincial perspective of the examined phenomena (Sarker et al., 2019). Thus, there are a lot to be accounted for regarding the social perspective as part of a wider spectrum of metaverses’ socio-technical continuum. Addressing metaverses in terms of both their social and technical aspects along a continuum, enables researchers and practitioners to enwidern their understanding of metaverses and address them in a broader intellectual and stakeholder base. Our analysis led to eight propositions of metaverses and to research questions which remain unanswered taking into account the role of various stakeholders as well as their activites within a metaverse. Hence, it inspires future research avenues while contributing to the recent discussion on metaverses within information systems and information management (Dwivedi et al., 2022). Given recent announcements of technology giants we expect the interest of the research community on this topic to be stimulated. To this end, we aspire that this paper could serve as an initial roadmap for future IS research on this timely, yet little understood topic.

Sheding light on metaverses’ business value provides insights on how the technology could serve as a driver of business innovation and change, thus the IT business value has been identified as one of the fundamental contributions of the IS field (Agarwal & Lucas, 2005; Kohli & Grover, 2008). While businesses are currently starting to invest in participating in the metaverse, their so far deployment and use of metaverses seems to be slower than initially expected. This might signal that we do not have enough empirical knowledge on how the metaverse shapes business value taking into account how the different stakeholders and activies shape business value of metaverse. In our analysis, we acknowledge that business value in the metaverse is rather complex and encompasses different stakeholders and levels of analysis. Table 3 provides several dimensions on the intersection of metaverse and business value regarding different levels, or can even be cross-level. Our suggestion is to consider the metaverse’s different levels of analysis concerning one or more activities depending on the research aim. For example, if the research aims to design ubiquitous metaverse experiences, researchers may need to go beyond one level of analysis or activities. Additionally, in Table 3, we note a close connection between the activities of strategy and tactics with measurement and value (they fall under the same propositions for each level of analysis).

This paper also holds practical implications. Recent investments of technology giants in metaverse-related applications have increased IT executives’ interest in further exploring metaverse’s potential for their organization. Indeed, businesses can identify a wide spectrum of metaverse applications. On the one hand, businesses can consider the metaverse a tool employed in the service of education, healthcare, social life, office life, etc. Furthermore, metaverse can lead to the creation of new tools for existing tasks or stimulate the conceptualization of new tools for new tasks and processes to support new or better solutions to existing challenges. Such digital innovations can lead to opportunities for business value generation, either within or outside of businesses. On the other hand, the metaverse can also serve as a target, offering the possibility to facilitate real estate applications, business transactions, games, or even role play (Dwivedi et al., 2022). Thus, this structured literature review can help IT executives in organizations better understand the potential of the metaverse and the potential value it can bring to businesses. Additionally, it can help organizations identify possible changes brought to their market and ecosystem of collaborators by the metaverse. Overall, metaverse applications can help us reimagine existing processes and change existing norms of how we work, behave, or interact with others.

### Limitations

The study suffers from some limitations. First, methodological limitations may affect the validity of this study’s findings. These limitations result from our choices in developing the review protocol and in the execution of the study. However, to some extent, we reduced such biases by searching all the major databases and following the steps indicated by Kitchenham and Charters (2007) and Dybå and Dingsøyr (2008). In addition, we only included articles that use the keyword metaverse, indicating that other relevant articles may exist which were not part of our analysis, such as articles on virtual worlds that do not mention metaverses (e.g., Nam et al., 2022; Zhang et al., 2020). To remedy this limitation, we conducted a backward-forward search (Webster & Watson, 2002) to manually identify potentially relevant articles. Second, we focused on papers of the last decade, aiming to shed light on the most recent work on this topic. Hence, our study is limited to papers published after 2012. Finally, the majority of papers reviewed focus on earlier versions of metaverses, which mainly relate to the use of virtual worlds and related technologies. We acknowledge that metaverses have evolved to incorporate cutting-edge technologies such as extended reality, human–computer interaction, artificial intelligence, blockchain, computer vision, edge and cloud computing, and mobile networks (cf. Section 1). In light of this evolvement, earlier interpretations of metaverses may inform the latest. Additionally, our study focused on metaverses and business value rather than social value. However, we acknowledge that metaverses may also hold social value (Dwivedi et al., 2022), which might impose indirect business value for organizations, e.g., sustainability (Papagiannidis & Bourlakis, 2010). To this end, the above limitations also suggest how researchers may conduct other types of literature reviews (Pare et al., 2015) or focus on outcomes beyond business value, such as social value or business transformation.

## Appendix

Table 4

**Table 4:** List of included primary studies in the systematic literature review

	Year	Authors	Title	Category	Journal / Conference
1	2017	Barin, A., Dolgov, I.,Toups, Z.O.	Understanding Dangerous Play: A Grounded Theory Analysis of High-Performance Drone Racing Crashes	Drones	Conference
2	2013	Bello-Orgaz, G., Camacho, D.	Comparative study of text clustering techniques in virtual worlds	User Behavior	Conference
3	2012	Amato, E., Sistach, C., Haute, L.	Inter Screen, between humans and avatars	Arts	Conference
4	2018	Sebastien, D., Sebastien, O., Conruyt, N.	Providing services through online immersive real-time mirror-worlds: The Immex Program for delivering services in another way at university	Education	Conference
5	2013	Vallance, M.	The affect of collaboratively programming robots in a 3D virtual simulation	Collaboration	Conference
6	2017	Webster, C., Garnier, F., Sedes, A.	Empty Room, an electroacoustic immersive composition spatialized in virtual 3D space, in ambisonic and binaural	Arts	Conference
7	2016	Ylipulli, J., Kangasvuo, J. Alatalo, T., Ojala, T.	Chasing Digital Shadows: Exploring Future Hybrid Cities through Anthropological Design Fiction	Urban Design	Conference
8	2015	Young, M.K., Rieser, J.J., Bodenheimer, B.	Dyadic interactions with avatars in immersive virtual environments: high fiving	User Behavior	Conference
9	2014	Herbet, A.	Immaterial art stock project: digital preservation in 3D virtual museum	Museums	Conference
10	2014	Kanematsu, H., Kobayashi, T., Barry, D.M., Fukumura, Y., Dharmawansa, A., Ogawa, N.	Virtual STEM Class for Nuclear Safety Education in Metaverse	Education	Journal
11	2015	Barry, D. M., Ogawa, N, Dharmawansa, A., Kanematsu, H., Fukumura, Y., Shirai, T., Yajima, K., Kobayashi, T.	Evaluation for Students’ Learning Manner Using Eye Blinking System in Metaverse	Education	Journal
12	2017	Choi, H., Kim, S.	A content service deployment plan for metaverse museum exhibitions—Centering on the combination of beacons and HMDs	Museums	Journal
13	2020	Suzuki, S., Kanematsu, H., Barry, D.M., Ogawa, N., Yajima, K., Nakahira, K.T., Shirai, T., Kawaguchi, M., Kobayashi, T., Yoshitake, M.	Virtual Experiments in Metaverse and their Applications to Collaborative Projects: The framework and its significance	Collaboration	Journal
14	2013	Crespo, R. G., Escobar, R.,F., Aguilar, L. J., Velazco, S., Castillo Sanz, A. G.	Use of ARIMA mathematical analysis to model the implementation of expert system courses by means of free software OpenSim and Sloodle platforms in virtual university campuses	Education	Journal
15	2014	Carneiro de Sousa, C.	The Meta_Body Project	User Behavior	Journal
16	2021	Liu, X., Zhang, J.	Foreign Language Learning through Virtual Communities	Education	Journal
17	2021	Tang, Y.	Help first-year college students to learn their library through an augmented reality game	Education	Journal
18	2013	Kanematsu, H., Kobayashi, T., Ogawa, N., Barry, D.N., Fukumura, Y., Nagai, H.	Eco Car Project for Japan Students as a Virtual PBL Class	Education	Journal
19	2021	Trkman, P., Černe, M.	Humanising digital life: Reducing emissions while enhancing value-adding human processes	User Behavior	Journal
20	2021	Barry, D.M., Kanematsu, H., Ogawa, N., McGrath, P.	Technologies for teaching during a pandemic	Education	Journal
21	2015	Badilla Quintana, M.C., Fernández, S. M.	A pedagogical model to develop teaching skills. The collaborative learning experience in the Immersive Virtual World TYMMI	Education	Journal
22	2012	Chandra, Y., Leenders, M. A.A.M	User innovation and entrepreneurship in the virtual world: A study of Second Life residents	Entrepreneurship	Journal
23	2018	Zhou, M. Cong, L.M., Leenders, M. A.A.M.	Ownership in the virtual world and the implications for long-term user innovation success	Entrepreneurship	Journal
24	2013	Kim, S., Joo, Y.S., Shin,M. Han, S., Han, J.	Virtual world control system using sensed information and adaptation engine	User Behavior	Journal
25	2012	Kim, C., Lee, S., Kang, M.	I became an attractive person in the virtual world: Users’ identification with virtual communities and avatars	User Behavior	Journal
26	2020	Balzerkiewitz, H., Stechert, C.	Use of Virtual Reality in Product Development by Distributed Teams	Collaboration	Journal
27	2019	Griol, D., Sanchis, A., Molina, J. M., Callejas, Z.	Developing enhanced conversational agents for social virtual worlds	User Behavior	Journal
28	2014	Jung, Y., Pawlowski, S.	Virtual goods, real goals: Exploring means-end goal structures of consumers in social virtual worlds	Marketing	Journal
29	2020	Chylinski, M., Heller, J., Hilken, T., Keeling, D. I., Mahr, D., Ruyter, K.	Augmented reality marketing: A technology-enabled approach to situated customer experience	Marketing	Journal
30	2015	Diego-Mas, J.A., Alcaide-Marzal, J.	A computer based system to design expressive avatars	Avatar Design	Journal
31	2014	Grant, S., Huang, H., Pasfield-Neofitou, S.	The Authenticity-anxiety Paradox: The Quest for Authentic Second Language Communication and Reduced Foreign Language Anxiety in Virtual Environments	Education	Journal
32	2014	Hassouneh, D., Brengman, M.	A motivation-based typology of social virtual world users	User Behavior	Journal
33	2013	Luse, A., Mennecke, B., Triplett, J.	The changing nature of user attitudes toward virtual world technology: A longitudinal study	User Behavior	Journal
34	2014	Papagiannidis, S., See-To, E., Bourlakis, M.	Virtual test-driving: The impact of simulated products on purchase intention	Marketing	Journal
35	2015	Ardila, L., Pérez-Llopis, I., Esteve, M., Palau, C.A.	Interoperable architecture for joint real/virtual training in emergency management using the MPEG-V standard	Emergency Management	Journal
36	2014	Sequeira, L.M., Morgado, L., Pires, E. J. S.	Simplifying Crowd Automation in the Virtual Laboratory of Archaeology	Archaeology	Journal
37	2016	Chen, J. C.	The crossroads of English language learners, task-based instruction, and 3D multi-user virtual learning in Second Life	Education	Journal
38	2012	Boughzala, I., de Vreede, G., Limayem, M.	Team Collaboration in Virtual Worlds: Editorial to the Special Issue	Collaboration	Journal
39	2012	O Riordan, N., Adam, F., O Reilly, P.	Innovation Co-Creation In the Digital World	Entrepreneurship	Conference
40	2017	Mütterlein, J., Hess, T.	Exploring the Impacts of Virtual Reality on Business Models: The case of the Media Industry	Entrepreneurship	Conference
41	2013	Bououd, I., Rouis, S., Boughzala, I.	Social loafing impact on collaboration in 3D virtual worlds: An empirical study	Collaboration	Conference
42	2012	Lattemann, C., Stieglitz, S.	Challenges for Lecturers in Virtual Worlds	Education	Conference
43	2012	Miller, A., Allison, C., Getchell, K.	Open Virtual Worlds: A Serious platform for Experiential and Game Based Learning	Education	Conference
44	2016	McKenna, B.,Vodanovich, S.	Digital Native Identity Development in Virtual Worlds	User Behavior	Conference
45	2020	Winkler, N., Roethke, K., Siegfried, N., Benlian, A.	Lose Yourself in VR: Exploring the Effects of Virtual Reality on Individuals’ Immersion	User Behavior	Conference
46	2020	Radianti, J., Majchrzak, T., A., Fromm, J., Wohlgenannt, I.	A systematic review of immersive virtual reality applications for higher education: Design elements, lessons learned, and research agenda	Education	Conference
47	2014	Chesney, T., Chuah, S., Hui, W., Hoffmann, R., Larner, J.	Determinants of Friendship in Social Networking Virtual Worlds	User Behavior	Journal
48	2014	Riedl, R., Mohr P. N. C., Kenning P.H., Davis, F.D.,Heekeren, H.R.	Trusting Humans and Avatars: A Brain Imaging Study Based on Evolution Theory	User Behavior	Journal

## Footnotes

1 https://decentraland.org/
